# Genome-wide expression quantitative trait loci (eQTL) analysis in maize

**DOI:** 10.1186/1471-2164-12-336

**Published:** 2011-06-30

**Authors:** Beth Holloway, Stanley Luck, Mary Beatty, J-Antoni Rafalski, Bailin Li

**Affiliations:** 1 DuPont Agricultural Biotechnology, Wilmington, DE 19880, USA; 2 Pioneer Hi-Bred International, Johnston, IA 50131, USA; 3 United Stated Department of Agriculture Honey Bee Breeding, Genetics, and Physiology Laboratory, Baton Rouge, LA 70820, USA

## Abstract

**Background:**

Expression QTL analyses have shed light on transcriptional regulation in numerous species of plants, animals, and yeasts. These microarray-based analyses identify regulators of gene expression as either cis-acting factors that regulate proximal genes, or trans-acting factors that function through a variety of mechanisms to affect transcript abundance of unlinked genes.

**Results:**

A hydroponics-based genetical genomics study in roots of a *Zea mays *IBM2 Syn10 double haploid population identified tens of thousands of cis-acting and trans-acting eQTL. Cases of false-positive eQTL, which results from the lack of complete genomic sequences from both parental genomes, were described. A candidate gene for a trans-acting regulatory factor was identified through positional cloning. The unexpected regulatory function of a class I glutamine amidotransferase controls the expression of an ABA 8'-hydroxylase pseudogene.

**Conclusions:**

Identification of a candidate gene underlying a trans-eQTL demonstrated the feasibility of eQTL cloning in maize and could help to understand the mechanism of gene expression regulation. Lack of complete genome sequences from both parents could cause the identification of false-positive cis- and trans-acting eQTL.

## Background

Genomic sequencing of crop species has shed light on causative relationships between sequence polymorphisms and traits of agronomic interest. Ongoing efforts in maize QTL (quantitative trait locus/loci) mapping have identified genetic intervals whose underlying genes variably contribute to interesting phenotypes such as oil content [1], root architecture [2], and pest resistance [3]. While many trait variations (quantitative and qualitative) result from amino acid differences [1,4], gene expression differences can also result in observable phenotypes [5]. Considering the burgeoning fields of epigenetics and transcriptomics, analysis of gene expression regulation is playing an important role in understanding gene interactions that lead to traits of interest.

The concept of "genetical genomics" [6] was proposed with the advance of high throughput gene expression profiling technologies. In traditional QTL analyses, linkage mapping leads to the detection of genomic regions which are associated with phenotypic variations within a population. Genetical genomics employs this same approach, except that the phenotypes are levels in gene expression resulting in the detection of expression QTL (eQTL). eQTL do not necessarily result from sequence polymorphisms proximal to the gene being measured (cis-acting) but could result from differences in genes unlinked to the target. In these cases, the eQTL function in a trans-acting manner.

The field of genetical genomics has allowed eQTL analysis within mapping populations in a multitude of species of plants [7-10], yeast [11,12], and mammals [13-16]. An *Arabidopsis *study [17] describes that while there are more trans-acting factors within the genome, cis-acting elements are more significant and therefore stronger in regulatory ability than those acting in trans. The results suggest a generalization that while multiple trans-acting factors can each weakly contribute to the total expression regulation of a given gene, a single cis-acting variation plays a far greater role.

The mapping and positional cloning of a trans-acting eQTL may reveal an expression regulator such as a transcription factor or small regulatory RNA. While many eQTL have been identified, few trans-acting factors have been cloned. Yvert et al. [12] mapped and cloned two yeast trans-eQTL that regulate genes known to be involved in pathways regulating pheromone response and daughter cell separation following budding. Interestingly, neither of the causative genes functions as expression regulators such as transcription factors nor through other expected mechanisms. However, their work suggests that the continued high-throughput eQTL studies will identify novel genes to better understand the regulation of known biochemical pathways. Master regulators such as LEAFY, an *Arabidopsis *regulator of at least seven components involved in reproductive development [18], were previously only identifiable through mutagenesis. Genome-wide trans-eQTL analyses may identify more master regulators that function to control several components of a single pathway.

We report a genome wide eQTL analysis in a highly utilized maize mapping population. We successfully identify both cis-acting and trans-acting genetic elements that cooperate to regulate gene expression in maize crown roots, and describe the pitfalls of detecting false cis- or trans-acting eQTL in the absence of perfect genomic sequences from both parents. In addition to this genome-wide analysis of regulating factors, we have positionally cloned a trans-acting factor.

## Results

### Global analysis of cis-acting and trans-acting eQTL in maize root

**Figure 1:**
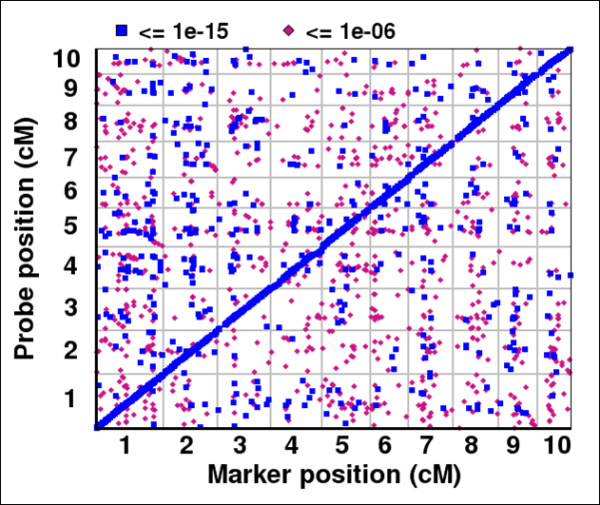
**Map position of eQTL**. Genetic position of the significant eQTL associations found in biological replicate 1. Genetic position of SNP marker (cM by chromosome) most significantly associated with the differential transcript abundance, x-axis. Genetic position of gene being measured by unique microarray probe, y-axis. The most significant associations (blue, KS p-value ≤ 1E^-15^) generally function in cis (diagonal line) where the eQTL and the transcript being measured map to the same genetic position. The weaker associations (pink, KS p-value ≥ 10^-15^) generally function in trans (periphery) where the eQTL maps to a position other than the transcript being measured.

The expression profile of the maize IBM2 Syn10 double haploid population [19] was determined by microarray hybridization to 60-mer probes. The mapping population was a subset of a population created through ten generations of intermating between the B73 and Mo17 maize lines followed by the generation of double haploid lines, which creates highly recombinant but fixed alleles. The more than 103,000 probes were designed to measure expression of the full complement of approximately 50,000 maize genes [20]. These expression level values were used as phenotypes for initial mapping analysis. While the array theoretically measures all genes, several factors must be considered: 1) the genomic origin remains unknown for a moderate number of genes; 2) probes may not be unique to individual genes but rather simultaneously measure several family members; 3) probes may not measure known genes, but rather expression originating from "non-genic" regions such as regulatory RNAs. Therefore, to ensure that appropriate mode of action can be assigned to eQTL, only those unique (as determined by BLAST analysis) genomic and/or genic probes were considered for eQTL analysis. Only the most significant eQTL was assigned for each probe, as described in Methods. A genetic distance window of 10 cM was used to define the mechanism of action for the eQTL. When the distance was less than 10 cM between the target gene and eQTL, the mechanism of action was considered cis-acting. Any eQTL greater than 10 cM from the target was deemed trans-acting. The filtering of eQTL by p-value significance, reproducibility between replicates, and confidence in unique probe hybridization eliminated nearly 90% of the probes from further analysis, resulting in 10,941 high quality eQTL. Of the most significant eQTL for each probe, the majority of eQTL analyzed functioned through a cis mechanism (9,795 of 10,941) and of those, 54% (5,311 of 10,941) functioned with a Kolmogorov-Smirnov (KS) p-value of ≤ 1E^-15^. Trans-acting eQTL were the minority of the eQTL identified (1,146 of 10,941), and of those 44% (500 of 1,146) functioned with a KS p-value of ≤ 1E^-15^. Altogether, these data suggest that cis-acting eQTL in general regulate gene expression with more significance than trans-acting eQTL (Table 1, Figure 1).

**Table 1:** Quantity and mode of action of significant (≤10^-6^) eQTL peaks mapping in biological replicate 1

	cis-acting	trans-acting
**KS p-value 10^-6 ^- 10^-15^**	4,484	646
**KS p-value 10^-15 ^- 10^-30^**	5,311	500

Significant eQTL (KS p-value ≤ 10^-6 ^) classified based on strength and trans- or cis-acting function for the 10,941 confirmed eQTL identified

### High reproducibility of biological replicates

**Figure 2:**
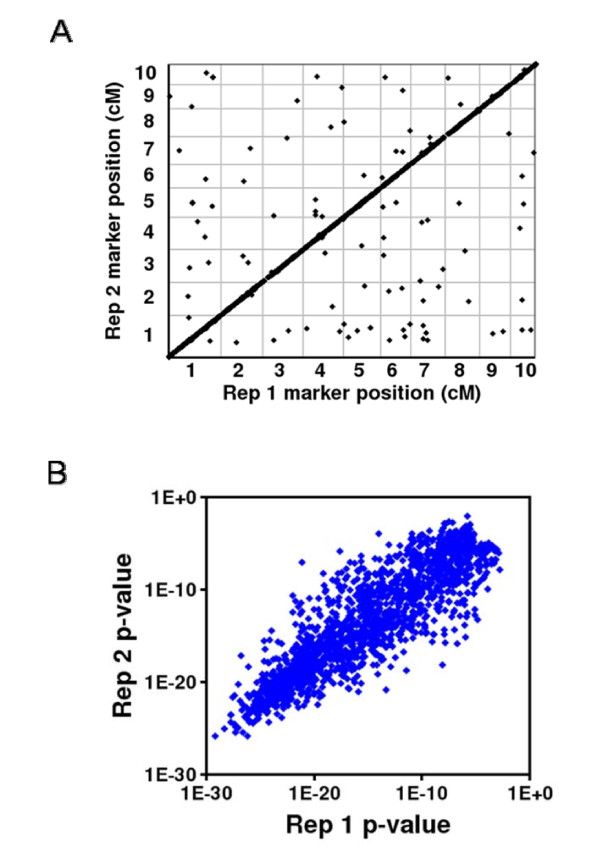
**Reproducibility of eQTL mapping between biological replicates**. A. Position of SNP marker most significantly associated for each validated eQTL determined for replicate 1 (x-axis) versus replicate 2 (y-axis). More than 99% of eQTL map to the same position across replicates. B. KS p-value determined for eQTL for replicate 1 (x-axis) versus replicate 2 (y-axis). The significance of each eQTL is reproducible across replicates. replicate 2 (y-axis).

The experimental design, utilizing a supported hydroponics system in growth chamber conditions, was intended to minimize false eQTL that resulted from environmental effects. While biological replicates were grown and harvested several months apart, the time and duration of harvest were comparable between replicates to minimize diurnal effects on transcription. Reproducibility between replicates was determined by the eQTL map position identified, as well as the confidence in the eQTL (p-value). Initially, eQTL were identified in the first replicate with a p-value cut-off of 1E^-6 ^(Additional File 1) but were validated in replicate 2 with a less stringent cut-off of 1E^-5 ^[21]. Based on high quality eQTL identified in replicate 1 alone, (n = 12,630), 86.6% (n = 10,941) were validated in replicate 2 and used for all subsequent global analysis. Those eQTL that failed to validate between replicates fell into two categories: 1) non-significant p-value in replicate 2 (12.5%, n = 1578) or 2) inconsistent map position (0.8%, n = 111) such that the position of the strongest eQTL failed to reproduce between replicates (Figure 2A). Further investigation of the 111 inconsistent eQTL suggests that the reasons for the inconsistency are complex and detailed analysis is required for appropriate determination of reproducibility. If several trans-eQTL in combination with a cis-eQTL contribute to expression control, it is possible that the eQTL deemed strongest in one replicate may be second strongest in another replicate, and vice versa. Additionally, if the eQTL peak broadly extends across a greater than the 10 cM interval, then significance variations between replicates may falsely suggest a failure of reproducibility for that eQTL. Therefore, the actual reproducibility might be slightly higher than the number indicated. For the eQTL that mapped reproducibly it is also important to note that the significance of each eQTL was consistent as well (Figure 2B). Altogether, this suggests that the hydroponics system and analyses enriched for the identification of genetic components of gene expression regulation.

### Cooperative regulation of gene expression

**Figure 3:**
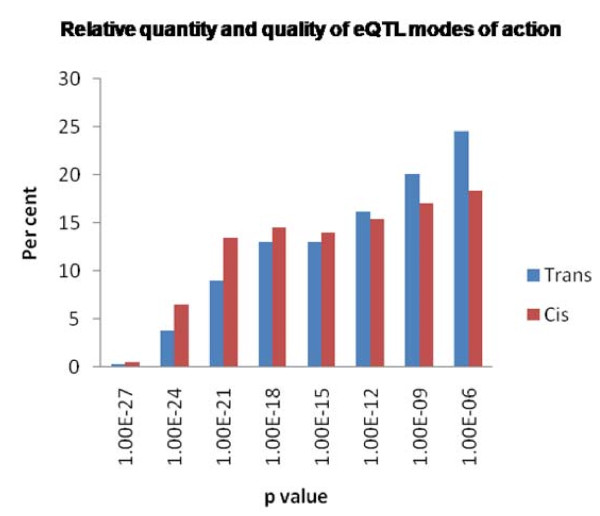
**Quantity and mode of action of eQTL**. eQTL were categorized as cis- or trans-acting based on map positions of the eQTL as compared to the genomic origin of the gene being measured by the microarray. A total of 10,941 eQTL were identified that were analyzable for mode of action based on strict transcript genomic origin criteria. Ninety per cent of all eQTL (9,795 of 10,941) functioned through a cis regulatory mechanism. Normalizing the number of eQTL identified as cis- or trans-shows that the stronger eQTL tend to function through cis-regulation (red, n = 9,795) while weaker eQTL tend to function through trans-regulation (blue, n = 1,146).

Previously published work [10,22] suggests that the vast majority of eQTL detected will function through a trans-acting mechanism (either individually or in combination), while the strongest (but fewer) eQTL will function through a cis-acting mechanism. However, a breakdown of strengths and quantities of each mode of action identified using the IBM2 Syn10 population showed nearly 9 fold more cis-acting than trans-acting eQTL, yet the cis- did function with greater significance (Table 1, Figure 3). There are several possible explanation for the discrepancy: 1) a conservative p-value cut-off of 1E^-6 ^is used to minimize the number of false positive eQTL identified, however, it is expected that many trans-acting eQTL would weakly regulate gene expression, perhaps with significance less than the cut-off value, and may be lost in the analysis; 2) Only the most significant eQTL was selected for each probe, which is more likely to be cis-acting (i.e. only a single, most significant eQTL is reported for each probe); 3) the microarray probes were strongly B73-biased (mostly designed from the B73 reference genome) and any imperfect hybridization of the Mo17 derived alleles to the probe set will map as cis-acting eQTL regardless of the genome-mediated functional expression regulation *in situ*; 4) The maize genome sequence is not completed. Many of the genomic origins of array probes remain unidentified or are not unique within the genome, therefore the transcripts being measured by the microarray cannot be accurately assigned to the current public genome assemblies, therefore the modes of action cannot be distinguished, potentially ignoring significant trans-eQTL; 5) A relatively large window (10 cM) was used to assign cis-eQTL potentially incorrectly characterizing eQTL.

A more comprehensive regulatory analysis can be performed in cases where multiple eQTL were identified for any particular target (Table 2). The general global gene regulation analysis identifies the most significant eQTL regulating any particular gene. Therefore, any secondary regulation of that gene is masked by the initial analysis. This second-level analysis enables the identification of regulatory elements that function with less significance than the primary eQTL that was initially identified. Of the eQTL analyzed, the vast majority (10,439 of 10,941) functioned independently to regulate target gene expression with the current statistic cut-off. Mapped genes that were regulated by 2 or more eQTL were further investigated. In total, 1,014 eQTL were detected for 502 target genes (Table 2). The majority of the initial primary (strongest) eQTL (79%, 395 of 502) mapped as cis. An additional 395 weaker secondary and 8 tertiary trans-acting peaks were found to regulate those same target genes. A minority (21%, 107 of 502) of initial primary eQTL mapped as trans-. An additional 74 secondary and 2 tertiary trans-acting eQTL were detected regulating those same target genes. Interestingly, 33 cis-acting eQTL were identified that function more weakly than the primary trans-acting eQTL. Using the stringent KS p-value cutoff of 10^-6^, the maximum number of cooperative eQTL identified for any particular gene was 3. This analysis demonstrates that although the strongest eQTL identified will likely function in cis, more trans-acting eQTL regulate gene expression, although with less significance.

**Table 2:** Cooperative expression regulation of 175 mapped genes

	cis-acting	trans-acting	total
primary eQTL	**395**	*107*	502

secondary cis-eQTL per primary eQTL	***n***	**33**	
secondary trans-eQTL per primary eQTL	*395*	*74*	
tertiary trans-eQTL per primary eQTL	*8*	*2*	

Total verifiable peaks identified	**428**	*586*	1014

The strongest eQTL identified regulating the expression of any particular gene is considered the primary identified eQTL. Up to two additional, yet weaker, functional expression regulators were identified regulating the same gene and are considered secondary or tertiary eQTL. Total verifiable cis-acting eQTL identified = 428 (**bold**); total verifiable trans-acting eQTL identified = 586 (*italics*). Due to the resolution of the genetic map, only one cis-acting eQTL can be identified per gene being measured, ***n ***= not possible.

### Lack of perfect genome sequences from both parental lines caused the identification of false cis- and trans-acting eQTL

**Figure 4:**
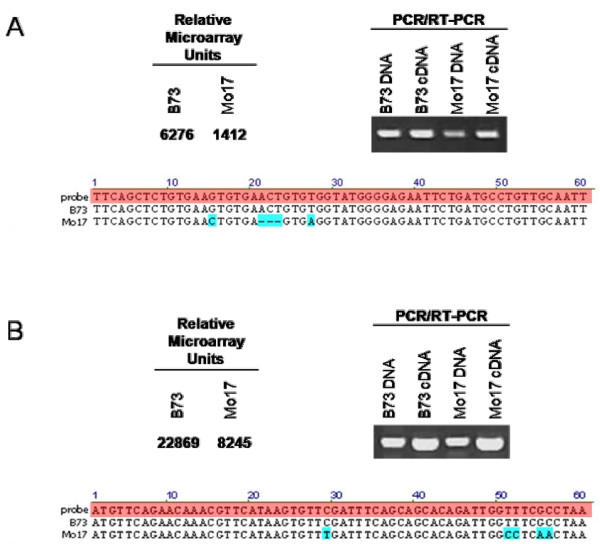
**False positive cis-eQTL**. Relative expression levels determined by the microarray suggests that cis-acting factors regulate the expression of two example genes. In both cases (A,B) the B73 transcript abundance is far greater than that of Mo17 according to the microarray. Amplification of genomic and cDNA sequence from B73 and Mo17 reveals that actual expression in situ may be more similar than the microarray would suggest. Sequence analysis of the probe-hybridization loci reveal that the microarray probes (red highlight) would fail to hybridize efficiently to the Mo17 alleles, thus inappropriately suggesting extreme differential expression and strong cis-eQTL. Blue boxes show polymorphisms between the Mo17-derived sequences as compared to the reference probe and B73-derived sequences.

While the B73 genome draft sequences are available, the Mo17 genome sequence is currently in a primitive state. Failure of Mo17 sequences to hybridize to their analogous probe due to sequence polymorphisms would map as cis-acting eQTL regardless of the actual expression levels or modes of action *in situ*. Of all the cis-eQTL, only 32.4% have higher expression levels for the Mo17 allele, which deviates significantly from the expected 50%. This implies that about 35% of the cis-eQTL are false positives. Of the most significant cis-eQTL (KS p-value ≤ 10^-25^), similar analysis indicates that 46% of them are false positives due to sequence polymorphism, To identify cases of such false positive eQTL, twenty of the strongest eQTL with unique genic/genomic probe positions in B73 were analyzed for sequence similarity in the Mo17 genome. BLAST of those probes to the publicly available paired-end read sequences of Mo17 generated by the Joint Genome Institute (JGI, US Department of Energy) found only two probes (10%) that match perfectly between B73 and Mo17 (data not shown). For the remaining eighteen B73 probes for which no identical Mo17 sequences were detected, it is not clear how many are caused by the incomplete Mo17 genome sequence and how many by sequence polymorphism. Using the B73 reference genome, primers were designed to amplify the intervals surrounding the probe hybridization sites for four of the potentially false-positive cis-eQTL. Two of the four intervals failed to amplify from Mo17 derived genomic and cDNA templates whereas they successfully amplified from B73 templates (data not shown). Without the Mo17 reference sequence, it remains unclear if polymorphisms prevented adequate primer annealing in Mo17 for PCR amplification. Two intervals amplified successfully from Mo17 template allowing for sequence analysis of the probe hybridization loci. Comparison of the amplified sequences from Mo17 to the B73 probe sequences showed that polymorphisms likely caused differential hybridization to the microarray probes (Figure 4). While trans- or cis-acting factors may function in the expression regulation of these genes *in situ*, the probe homology issues inappropriately suggest strong cis-eQTL. Therefore these strong cis-eQTL are most likely false positives.

**Figure 5:**
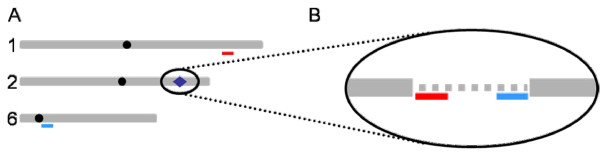
**Sequence gap in the physical map masks probe homology sites**. A Schematic of selected maize chromosomes. A "master regulating" trans-eQTL residing on chromosome 2, BAC AC207404 (blue diamond) regulates expression of two iron superoxide dismutase genes on the long arms of chromosomes 1 (BAC AC221053) and 6 (BAC AC187242), as measured by unique microarray probes (red and cyan, respectively); centromeres = *black circles*. B Re-sequencing analysis of the eQTL interval on chromosome 2 revealed a 2 kb gap (gray dotted line) in the known sequence (gray bars). Probe homology sites for both regulated genes were discovered within the gap negating a trans-acting master regulator effect, therefore suggesting a strong cis-acting eQTL in the vicinity of the newly identified third iron superoxide dismutase gene.

Despite the availability of the draft B73 genome sequences, incompleteness and inaccuracy in genome/BAC sequencing or gene annotations remain problematic for eQTL analysis. Additionally, the ancestral genome duplication in maize complicates mapping analysis when markers, genes, and genomic regions are indistinguishable. We identified a strong eQTL (KS p-value of 1E^-18^) that functioned apparently in trans- to regulate two iron superoxide dismutase (FeSOD) genes. Initially the eQTL was regarded as a master regulator considering its ability to regulate the expression of multiple biochemically related genes. Importantly, BLAST analysis of the microarray probes suggested that they measured their respective FeSOD transcripts uniquely. However further analysis of the eQTL interval and sequencing of the single BAC that spanned the interval revealed a sequencing gap in the available B73 genomic sequences. Within that gap resided a third FeSOD gene which the probes would recognize during microarray hybridization (Figure 5). Rather than identifying a strong trans-acting master regulator, most likely we have inadvertently revealed an unknown gene that was differentially expressed between B73 and Mo17 due to cis-acting factors, yet had high sequence similarity to other known FeSODs.

It is obvious that some of the cis- and trans-acting eQTL identified are false due to the unavailability of complete genome sequences for both parental lines. It is not clear how extensive the problem is, although it seems the strongest eQTL are more prone to be false positive than the weaker ones.

### Differential gene expression confirms presence/absence variations

In addition to nucleotide polymorphisms or minor insertion/deletion differences between the B73 and Mo17 parent genomes, several studies have identified large deletions in the Mo17 genome as compared to B73 [23,24]. Of particular interest is a deletion spanning many BACs on the short arm of chromosome 6 which contains at least 23 genes and pseudogenes [23]. eQTL mapping using the IBM2 Syn10 population found 28 strong (KS p-value range = 10^-21 ^- 10^-10^) cis-acting eQTL, measuring 28 probes (from 26 annotated genes) located on the BACs in question. For the genes in the region, B73 in general shows robust expression while Mo17 expression is essentially off (data not shown). The eQTL results are consistent with the presence/absence variations (PAV) detected through array genome hybridization.

### Identification of a trans-acting regulator by map-based cloning

**Figure 6:**
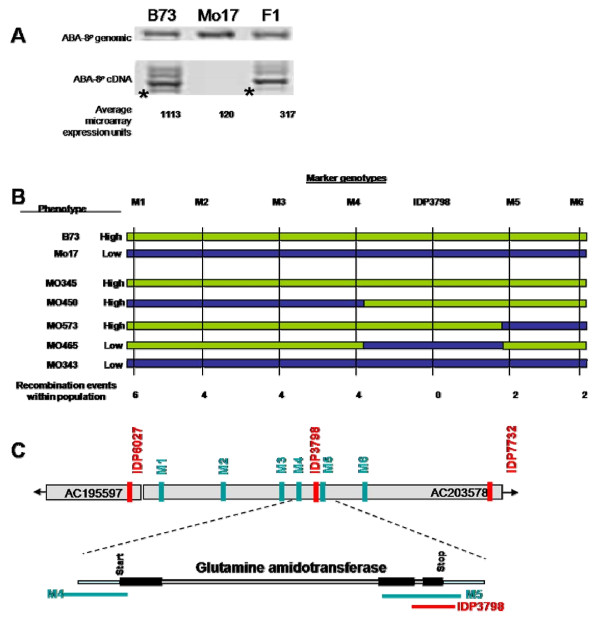
**Fine mapping of eQTL regulating ABA 8'-hydroxylase**. A Microarray expression data revealed greater expression of the target gene ABA 8'-hydroxylase. The target gene is present in both parental backgrounds but is much more strongly expressed under control of a B73 derived allele of the trans-regulator glutamine amidotransferase. Multiple splice variants are amplified from the pseudogene cDNA. Sequence analysis of variants shows that only the smallest band is able to hybridize to the microarray probe, *asterisk*. Among parental controls, the B73 inbred average expression shows a 9-fold greater expression of the pseudogene as compared the Mo17 inbred. An F1 hybrid shows mid-level expression regulation of the relevant band, *asterisk*, as well as several others. B Example phenotypes/genotypes found within the IBM2 Syn10 population and controls. Phenotypes are considered "high" or "low" depending on the relative ABA 8'hydroxylase target gene expression levels. B73 alleles of the eQTL allow for high expression of the target as compared to Mo17 alleles; B73 derived allele = green, Mo17 derived allele = blue. Fine mapping of the IBM2 Syn10 population using molecular markers (M1-M6) identified a very small region at the 3' end of the gene as being the responsible element that determines the expression of the target. **C **BACs within eQTL interval with public markers (IDP markers; red) and designed mapping markers M1-M6 (teal) spanning the region (not drawn to scale). IDP3798 and two mapping markers are located within the glutamine amidotransferase gene.

**Figure 7:**
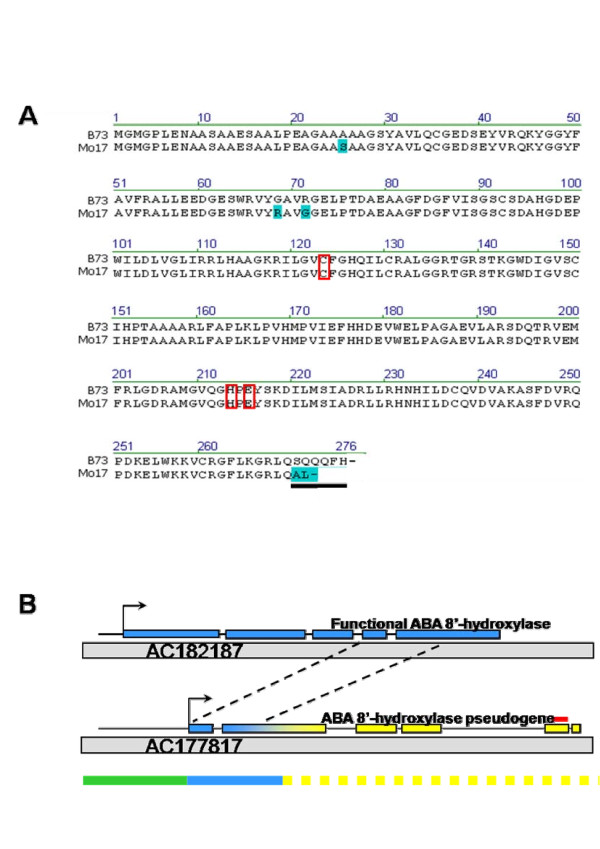
**Unexpected expression regulation of a pseudogene results from sequence variation in the carboxy terminal of glutamine amidotransferase**. A The B73 and Mo17derived sequences for the trans-regulator glutamine amidotransferase code for nearly identical proteins. Blue boxes highlight residues different from the B73 reference sequence. Fine mapping of the eQTL to a 186 bp interval determined that the carboxy-terminus accounts for the trans-regulation, *black bar*. Class I glutamine amidotransferase proteins require the conserved C-H-E triad (red boxes) for their expected enzymatic functions suggesting that, despite the sequence differences between the genotypes, the proteins likely remain functional in their expected pathway *in situ. *B Genomic structure on chromosome 1(BAC AC177817) that encodes for the pseudogene is derived from chimerization of 1.5 exons of the functional ABA 8'-hydroxylase genic region (blue bar, blue exons) of chromosome 4 (BAC AC182187), a 5' element enabling transcription originating from chromosome 10 (green bar; BAC AC194847), and genomically non-unique sequence (yellow dashed bar, yellow exons). The microarray probe (red bar) used to measure the pseudogene is unique within the transcriptome.

A strong trans-acting eQTL was selected for fine mapping and positional cloning to identify the gene responsible for expression regulation. We identified a strong eQTL (KS p-value < 1E^-26^) located on the long arm of chromosome 1 ((approximately 864 centimorgans (cM) on IBM2 Neighbors map [25])) that regulated the expression of a gene annotated as ABA 8'-hydroxylase, located 360 cM away from the eQTL near the centrosome of chromosome 1 (approximately 505 cM on IBM2 Neighbors Map). Within the mapping population, a B73 derived allele at the eQTL resulted in 6-fold greater expression (9-fold greater in parental controls) of the target gene as compared to a Mo17 derived allele at the eQTL (Figure 6A). The recombinant individuals from the IBM2 Syn10 population grown for the global eQTL analysis were used to define the genetic interval to within 186 base pairs in a single gene (Figure 6B, Additional File 2). The candidate gene underlying the eQTL was determined to be a class I glutamine amidotransferase domain containing gene (Figure 6C). Sequence analysis of the B73 and Mo17 alleles of the full length gene revealed multiple SNPs coding for five amino acid residue differences between the parents plus a 4 residue truncation in Mo17 as compared to B73 (Figure 7A). Only the 4 residue truncation is located within the 186 base pair eQTL interval, thus presumably the causative variation for the differentially gene expression. Re-phenotyping the IBM2 Syn10 population by RT-PCR specific for ABA 8'-hydroxylase confirmed the gene expression results from the microarray analysis.

Glutamine amidotransferase genes are well characterized players in several biosynthesis pathways including the purine, pyrimidine, histidine, tryptophan, and arginine pathways [26], however, a transcript-abundance mediating function has never been published. While steady state transcript abundance is determined by many factors including transcription factors, enhancers, mRNA degradation regulators, and rate of translation [27], the role of glutamine amidotransferase in ABA 8'-hydroxylase transcript abundance remains elusive. Despite the role that glutamine amidotransferase plays on ABA 8'-hydroxylase transcription, no other eQTL mapped to the same genetic region in this population suggesting that any broad transcriptional regulatory function is unlikely.

eQTL are determined by expression phenotypes, and not based on physiological or morphological phenotypes as in classic QTL identification. Therefore, further analysis of ABA biosynthesis in the IBM2 Syn10 population could prove a physiological function downstream of transcript abundance in regards to its trans-regulation. In anticipation of ABA biosynthesis analysis, the ABA 8'-hydroxylase gene being regulated by glutamine amidotransferase was sequenced from B73 and Mo17 cDNA. Interestingly, the results showed that the annotated ABA 8'-hydroxylase gene was actually a product of genomic shuffling that occurred sometime prior to the genetic diversification of B73 and Mo17. In both parentages, the ABA 8'-hydroxylase pseudogene (ABA-8'^*p*^) is a chimera of the functional maize ABA 8'-hydroxylase gene fragment, non-genic genomic sequence originating from chromosome 10, and repetitive genomic sequence (Figure 7B). Thus it seems that a pseudogene expression level is regulated. The ABA 8'^*p *^is not present in all maize inbred lines. Actually, validation of glutamine amidotransferase as the candidate gene for the eQTL was hampered by the absence of this pseudogene in certain lines (data not shown). Whether this pseudogene or its expression regulation has any physiological function remains to be determined.

## Discussion

While the field of genomics has afforded scientists with access to genomic sequences of countless numbers of model species, strains, and lines, our understanding of the function of those sequences remains rather limited. Forward and reverse genetics have given meaning to sequence polymorphisms in a fair number of genes, but in addition to gene function analysis, it is equally important to understand how and when those genes are activated and the roles the translated proteins play within biochemical pathways. Only through the continued efforts in the fields of transcriptomics and proteomics can the full power of genomics be realized. Expression QTL studies are generating vast amounts of data from the perspective of gene regulation, both from cis-acting elements and trans-acting factors, that begin to fill in gaps in the understanding of transcriptional regulation and gene interactions. Results from our study help to elucidate the genome-wide expression regulation in play during the development of crown root tips in maize. Despite stringent statistical analysis, we identified more than 10,000 eQTL that function through both cis- and trans-acting mechanisms. In addition to the identification of cis- and trans-factors, we described the relative regulatory contribution each of those factors plays by means of a KS p-value. Despite our expectations, as well as those set forth in previously published maize eQTL studies [28,29], we identified vastly more cis-acting eQTL than trans-suggesting that the most significant eQTL will act in cis for most genes. The statistical methodology and stringency we employed was designed to minimize false positive eQTL from the analysis, however at the probable expense of the lesser significant eQTL which will most likely function in trans [22]. Additionally, the definition of trans can be "arbitrarily" set for each study. While we defined trans-acting to mean any regulatory element greater than 10 cM from the target, others have set the boundary at 5 cM [29]. Either limit is appropriate for maize eQTL studies but will affect the classification and quantification of trans-eQTL.

Among the tens of thousands of cis- and trans-acting eQTL identified in the current study, we have demonstrated that some of them are false-positives due to the lack of complete genome sequences from both parents of the mapping population. Polymorphism in the microarray probe regions, which could affect hybridization intensities for mRNA quantification, will lead to the occurrence of false cis-acting eQTL. Likewise, sequencing gaps at the trans-acting eQTL regions could result in the detection of false trans-acting eQTL. With these mechanisms, the stronger, large-effect eQTL (with low p-values) are more prone to be false positives than the weaker eQTL. We estimated that 35% of all the cis-eQTL is false positive, but the false trans-eQTL discovery rate is unknown in the absence of the complete genome sequences. It is likely that the whole-genome eQTL analysis reported in other systems could suffer similar false positive issues, and caution should be taken when interpreting the results and selecting eQTL for further analysis.

Although tens of thousands of eQTL have been reported, very few have been cloned and characterized, especially in higher organisms where positional cloning could still be challenging. To test the feasibility of eQTL cloning in maize and to understand the regulatory mechanism of transcript abundance, a strong trans-eQTL for a putative ABA hydroxylase was selected for further mapping and cloning. The eQTL was fine mapped to a very small physical interval (186 bp) and a putative glutamine amidotransferase gene was identified as the candidate gene for this eQTL. Unfortunately, the target gene identified turned out to be a pseudogene. It is not clear if this ABA hydroxylase pseudogene has any physiological function, why the expression of a pseudogene is regulated, or how the two genes interact with each other. Nevertheless, we have clearly demonstrated the feasibility of cloning trans-eQTL with large effect in maize. The cloning of trans-eQTL would help to understand the mechanism of transcript abundance regulation and identify regulatory genes for biochemical pathways.

The IBM2 SYN10 population is a set of 360 doubled haploid lines from a randomly mated population derived from B73 and Mo17 [19]. Having undergone 10 generations of random intermating/recombination, these DH lines exhibited a high degree of phenotypic variability and high frequency of recombination. We were able to fine map an eQTL into a 186 bp interval with only 135 IBM2 SYN10 DH lines. Although recombination frequency varies widely across the genome and more genomic loci need to be analyzed before this population can be better assessed for recombination frequency, the results suggest that the IBM Syn10 population is suitable for high resolution eQTL mapping and recombination studies.

As in previous reports, we have shown that most of the strong eQTL act in cis. Moreover, for the relatively small number of strong trans-eQTL detected, some of them could be false positive. Therefore, it may be a challenge to identify a large number of strong trans-eQTL which are more amenable for positional cloning. A recently study in human B cells which maps the regulatory elements that influence radiation-induced changes in gene expression indicated that nearly all the strong regulators act in trans to influence the expression of their target genes [30]. Therefore, instead of mapping the steady-state level of mRNA under one constant condition, mapping eQTL which regulate the differential responsiveness in gene expression to biotic and abiotic factors could be a promising approach to enrich and identify strong trans-eQTL. These trans-eQTL should be ideal targets for cloning. They are important for understanding plant responses to biotic and abiotic stresses, and technically feasible to isolate.

## Conclusions

We have shown the feasibility of eQTL analysis as a means to identify, clone and analyze trans-acting regulatory factors through large-scale screening analysis. A glutamine amidotransferase regulator was identified as a trans-acting factor. Harnessing the regulatory function of trans-acting factors could allow for better control of important agronomical genes. We also described the pitfall of identifying false-positive eQTL in the absence of complete genome sequences, which has broad implications in similar eQTL studies.

## Methods

### Hydroponic system

Plants were germinated and grown in Turface ^®^MVP^® ^contained in Deepot™ plastic conical pot and tray systems (Stuewe and Sons, Tangent, OR (D25L [pots], D50T [trays])). The hydroponic system consisted of stainless steel tanks (24"w × 24"d × 15"h) with a bottom-filling pumping and drainage system and overflow drain at the level of the top of the tubes/Turface^®^MVP^®^. Seeds were planted approximately 2 cm below the top of the tube/Turface^®^MVP^® ^and germinated for one week in diH_2_O submerged so that the seeds were just above the water line. After germination, the water was drained and a media pumping system was initiated. A humidity cover was in place from the initial germination until plants were approximately 6 inches tall. Modified Hoagland's Solution (1 mM KH_2_PO_4_, 2.5 mM KNO_3_, 1.25 mM Ca(NO_3_)_2_, 1 mM MgSO_4_, 0.75 mM CaCl2, SPRINT330 such that 0.006 mM with respect to Iron, 0.03 mM FeSO_4_, 1 μM H3Bo_3_, 1 μM MnCl_2_, 1 μM ZnSo_4_, 1 μM CuSO_4_, 1 μM NaMoO_4_) was pumped into the tanks until the tubes were submerged but the vegetative growth remained dry. The nutrient solution remained in the tank for approximately 3 minutes before draining. The submersion/drainage system repeated every 3 hours for the 3 weeks following germination. Growth chamber conditions were maintained at 50% humidity with a 16 h day (26°C) and an 8 h night (22°C). Light levels ranged from approximately 350 μM/m^2^/s at the base of the plant to 500 μM/m^2^/s at the top of the leaves at the time of harvest.

### Plant materials and tissue preparation

For each of two biological replicates, tissue was collected from B73 and Mo17 parental controls and the IBM2 Syn10 doubled haploid mapping population [19] at 4 weeks after germination (V5 stage). The IBM Syn10 population consisted of 360 doubled haploid lines resulting from 10 generations of intermating, followed by a double haploid process creating highly recombinant, yet fixed alleles. One hundred thirty-five IBM2 Syn10 lines were used in this study. The tissue sampled was the 1.5 cm tip of all crown roots that had developed at the time of harvest, representing the most metabolically active region based on whole-tissue visualization of triphenyl tetrazolium chloride staining [31]. Tissues were flash frozen in liquid nitrogen and stored at -80°C. They were manually ground into a fine powder using a mortar and pestle on dry ice.

### Microarray hybridization

Total RNA was isolated from frozen ground tissue (SQ Tissue Kit, Omega Biotek) and treated with DNase-I followed by polyA RNA isolation (Illustra mRNA Purification Kit, GE Biosciences) for all samples. The total RNA and polyA RNA samples were visualized and quantified on Agilent's Bioanalyzer to check for degradation and to determine the concentration. Each mRNA sample was made into double stranded DNA, amplified by an in-vitro transcription reaction and labeled with Cy3 or Cy5 fluorescent dyes using Agilent's Low RNA Linear Amp Kit. The cRNA product was purified with Agencourt's RNAClean Kit that utilizes SPRI (Solid Phase Reversible Immobilization) paramagnetic bead-based technology. Overnight hybridizations were performed with equal amounts of labeled cRNA to custom 2 × 105K [20] Maize Oligo Microarrays from Agilent Technologies (Palo Alto, CA) according to Agilent's Two-Color Microarray-Based Gene Expression Analysis protocol. After hybridization, the microarray slides were washed and immediately scanned with Agilent's G2505B DNA Microarray Scanner at two laser power settings (100% and 10%). The images were visually inspected for image artifacts and feature intensities were extracted, filtered, and normalized with Agilent's Feature Extraction Software (v 9.5.1). Further quality control analysis was performed using data analysis tools in Rosetta's Resolver Database. The Agilent microarray expression data (raw and processed) are available from GEO (Series Accession GSE29964).

### Statistical analysis and eQTL mapping

Microarray intensity data were determined from each of the two channels using the Rosetta Resolver Split-Ration method [32,33], were exported from the Rosetta Resolver Database and analyzed using software developed in-house. The following is a brief summary of the concepts that underlie our data analysis methods. A multidimensional, weighted least-squares method was used to obtain normalization parameters for the data based on affine transformations, an effective normalization approach according to measurement theory [34] and fluorescence instrumentation considerations [35]. A further implication is that various stochastic effects, both instrumental and biological, give rise to an overall intensity dependent noise [36-38]. Data was analyzed using the intensity representation to preserve the noise characteristics and estimate statistical significance, facilitating the use of linear least-square methods for data analysis. The intensity distribution shows that about 20 percent of the data from a microarray typically corresponds to background. Therefore an additive correction was applied to adjust the average background signal for a microarray to zero, prior to normalization. As described above, gene expression was measured using a single color experiment design for two biological replicates of the SX19 Syn10 population. The population was also genotyped at 1731 SNP markers using the Illumina assay. Initially, 50 eQTL were individually mapped using the Knott-Haley regression method for interval mapping (Windows QTL Cartographer, Statistical Genetics and Bioinformatics, North Carolina State University, USA) to confirm subsequent analyses where the Kolmogorov-Smirnov (KS) test was used to test each marker for association with normalized intensity from each gene signature. The map positions of eQTL were assigned based on the most significant KS p-value calculated for each probe by genome wide association scanning, therefore assigning only one eQTL per probe. Various methods have been proposed for controlling the false positive rate on the basis of uniform distributions of p-values [39,40]. However, the problem of adjusting for bias arising from correlation in hypotheses remains a challenge [41,42]. Thus, we adopt a heuristic approach based on the analysis of the distribution of KS p-values for all eQTL, which gives a threshold of 10^-5 ^for controlling the false discovery rate. Furthermore, association testing methodology including those used here rely on assumptions that may not hold, particularly with regard to noise. Therefore, a slightly more conservative threshold of 10^-6 ^was used for identifying eQTL, and to obtain a lower false positive rate for the purposes of this study. To test overall reproducibility, data for each biological replicate were analyzed separately for eQTL including eQTL map position, KS p-value, and relative microarray intensity.

### Map-based cloning of a trans-acting regulator

A strong trans-acting eQTL was selected for fine mapping and cloning. Genomic DNA from the IMB2 Syn10 population and parental controls were purified from leaf tissue using Gentra^® ^Puregene^® ^(Qiagen, Valencia, CA) [43] modified in scale of preparation. Fine mapping of the interval was initiated at the genetic position determined by eQTL mapping analysis of the microarray expression data. Total RNA for gene expression validation was purified as above and reverse transcribed into cDNA using QuantiTect^® ^Reverse Transcription Kit (Qiagen, Valencia, CA). The B73 and Mo17 alleles of the candidate gene, glutamine amidotransferase, were amplified from cDNA using the primers 5'-CCTAAGACATCCCAATTTCCTC and 5'-GTCGCCTCCATCTCCATTC, cloned into the pCR^®^2.1.TOPO^® ^TA vector (Invitrogen, Carlsbad, CA), and were confirmed by sequencing. The eQTL was re-mapped using the IBM2 Syn10 population based on a quantitative RT-PCR phenotype for the target previously measured by the microarray (a pseudogene of ABA-8'Hydroxylase) using the primers 5'-GCGTTGAACACTTGGACCAC-3' and 5'-TGGAAGGTGTTGCCCCTGTT-3'.

## Authors' contributions

BH carried out the sample collection and processing, molecular analyses including fine mapping, cloning, and laboratory-based confirmation of microarray results, and drafted the manuscript. SL performed the statistical normalization analysis of microarray results and large scale mapping of eQTL. MB performed the microarray expression profiling experiments. JAR and BL conceptualized and coordinated the project. All authors read and approved the final manuscript.

## Supplementary Material

Additional file 1 **Controlling the false discovery rate**. The cumulative count of gene expression probes is plotted against KS p-value for the most significant eQTL for each probe on a logarithmic scale. The heavy tail to the left is associated with the eQTL that are most significant overall. The right side of the cumulative curve is associated with the least significant eQTL; i.e. the noise. The nonlinear regression curve shows that the noise is characterized by a power-law with exponent of 0.24; a uniform distribution would have exponent 1.0. The p-value data deviates from the fitted curve at 10^-5 ^which serves as a threshold for overall significance for eQTL. The distributions of p-values from eQTL scans for individual probes also show deviation from uniformity. The non-uniformity arises because the statistical tests for markers are not independent due to genetic linkage. Click here for file

Additional file 2 **Molecular markers designed for fine mapping the eQTL regulating the ABA 8'-hydroxylase pseudogene**. Primers designed to define the interval containing the eQTL. All primers are written 5' to 3'. Restriction enzyme digestion is required for 4 of the markers to visualize the polymorphisms. Click here for file
