# Supplementary figures and images for: Genome-wide expression quantitative trait loci (eQTL) analysis in maize
Source: BMC Genomics. 2011 Jun 30;12:336. doi: 10.1186/1471-2164-12-336 (PMC3141675; doi:10.1186/1471-2164-12-336)

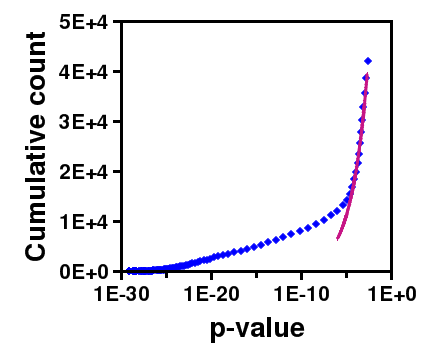

Supplement: Additional file 1 — Controlling the false discovery rate. The cumulative count of gene expression probes is plotted against KS p-value for the most significant eQTL for each probe on a logarithmic scale. The heavy tail to the left is associated with the eQTL that are most significant overall. The right side of the cumulative curve is associated with the least significant eQTL; i.e. the noise. The nonlinear regression curve shows that the noise is characterized by a power-law with exponent of 0.24; a uniform distribution would have exponent 1.0. The p-value data deviates from the fitted curve at 10-5 which serves as a threshold for overall significance for eQTL. The distributions of p-values from eQTL scans for individual probes also show deviation from uniformity. The non-uniformity arises because the statistical tests for markers are not independent due to genetic linkage. [file 1471-2164-12-336-S1.TIFF]

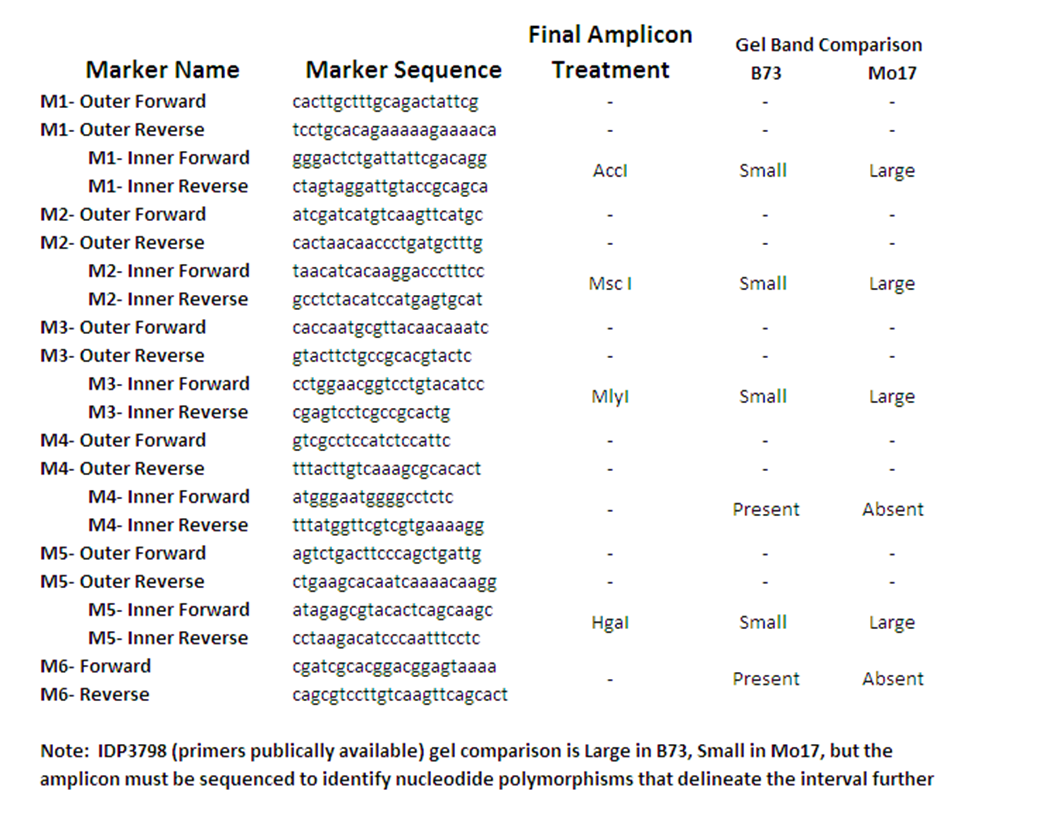

Supplement: Additional file 2 — Molecular markers designed for fine mapping the eQTL regulating the ABA 8'-hydroxylase pseudogene. Primers designed to define the interval containing the eQTL. All primers are written 5' to 3'. Restriction enzyme digestion is required for 4 of the markers to visualize the polymorphisms. [file 1471-2164-12-336-S2.TIFF]
